# Reporting guidelines for realist evaluations seek to improve clarity and transparency

**DOI:** 10.1186/s12916-016-0658-7

**Published:** 2016-07-21

**Authors:** Vivian A. Welch, Andrea C. Tricco

**Affiliations:** Bruyère Research Institute, Ottawa, Ontario Canada; School of Epidemiology, Public Health and Preventive Medicine, University of Ottawa, Ottawa, Ontario Canada; Li Ka Shing Knowledge Institute, St. Michael’s Hospital, Toronto, Ontario Canada; Epidemiology Division, Dalla Lana School of Public Health, University of Toronto, Toronto, Ontario Canada

**Keywords:** Realist evaluation, Reporting guidelines, Knowledge synthesis

## Abstract

An increasing number of realist evaluations are being conducted from a wide range of disciplinary perspectives and with diverse, fit-for-purpose methods. This commentary discusses the recent BMC Medicine publication of RAMESES II reporting guidelines for realist evaluations. Knowledge users such as program implementers and decision-makers will benefit from the increased transparency of reporting and interpretation in light of the totality of evidence encouraged by this guidance. It is hoped that these reporting guidelines will eventually lead to improved knowledge synthesis and contribute to the cumulative science regarding realist evaluation.

Please see related article: https://bmcmedicine.biomedcentral.com/articles/10.1186/s12916-016-0643-1

## Background

Realist evaluations are theory-driven evaluations that seek to understand how complex interventions work, for whom they work, and how programs and their effects are influenced by the context and a setting that is rooted in the philosophy discipline [1, 2]. Realist evaluations are carried out from different disciplinary perspectives and using a plurality of methods that are fit for purpose. They can be useful in understanding how a program or policy works, in which settings, and for whom. Realist evaluations can be used to hypothesize whether the program works in different settings and for different participants, including “program designers, implementers, and recipients” [3]. The hypotheses are tested and refined during the evaluation of the program, and this evaluation can be understood using the context-mechanism-outcome (CMO) configuration. Because of this, realist evaluations are appreciated by implementers and decision-makers who seek to understand how a program or policy works, and in which circumstances, when designing or funding programs.

In a recent article in *BMC Medicine*, Wong and colleagues seek to improve the transparency of reporting realist evaluations by developing consensus and evidence-based reporting guidelines for realist evaluations [3]. They use transparent and accepted methods endorsed by the EQUATOR Network [4] and outlined by Moher and colleagues [5] to develop this guidance. Their protocol was published in *BMJ Open* [6] and 35 experts with diverse disciplinary backgrounds and experience from six different countries participated in three rounds of a Delphi survey to develop this guidance. A high response was achieved across all rounds of the Delphi (range 76–90 %).

The tool consists of 20 items, which have been broken down into the following six sections: Title, Summary of Abstract, Introduction, Methods, Results, and Discussion. Each of the 20 items included in their reporting realist evaluations tool includes a detailed rationale for each item and exemplars of good practice. They have also built in flexibility in terms of the order of reporting and they strongly encourage authors to document a justification for any variance from the reporting items, including omissions of items. The first item is related to identifying the study as a realist evaluation in the title, which will aid in identifying these types of studies in the future. Items 3 (rationale for evaluation), 4 (program theory), 7 (rationale for realist evaluation), 8 (environment and context), and 10 (evaluation design) are particularly important because, based on evaluations of the reporting of realist reviews [7], these items are more likely to be poorly reported. Items 11 through 13 relate to data collection, the recruitment process, and data analysis. Item 16 on the summary of findings encourages authors to rate the strength of the evidence from the evaluation, which is extremely important for stakeholders who seek to use this information. Item 20 relates to the source of funding and declaring any potential conflicts of interest.

One major advance of this reporting guideline is the encouragement to situate the realist evaluation in the totality of the evidence (item 18), which will help program implementers to interpret the findings in light of other relevant evidence while considering the contribution of differences in settings and populations. This is in keeping with other global initiatives to consider the entirety of the evidence when reporting results of primary studies, such as *The Lancet* guidelines [8] and the CONSORT Statement [9]. We agree with the authors that situating findings in the light of relevant evidence will contribute to the cumulative evidence base and science regarding other similar programs and policies.

The authors are already promoting the uptake of these reporting standards through the RAMESES listserv, and training workshops and materials, which will assist in their uptake by program evaluators. Also, the authors plan to evaluate the usefulness and impact of these reporting guidelines in the future. An additional activity that could enhance the impact of these reporting guidelines is registration with the EQUATOR Network [4]. Also, the EQUATOR Network provides tools and resources for journal editors to facilitate the use of reporting guidelines by authors.

## Conclusions

The RAMESES II reporting guidelines for realist evaluations is an important initiative to ensure these evaluations are reported in sufficient detail, in the context of existing evidence, and with a rating of strength of evidence for main findings that will greatly assist users of the evaluations. Because reviews are only as good as the included studies, in our opinion, this could also eventually improve realist syntheses that include realist evaluations [10]. We look forward to the upcoming development of quality methodological standards for realist evaluations, which will also advance the science of this type of study, as well as likely improve realist synthesis.
